# Identification of a drug binding pocket in TMEM16F calcium-activated ion channel and lipid scramblase

**DOI:** 10.1038/s41467-023-40410-x

**Published:** 2023-08-12

**Authors:** Shengjie Feng, Cristina Puchades, Juyeon Ko, Hao Wu, Yifei Chen, Eric E. Figueroa, Shuo Gu, Tina W. Han, Brandon Ho, Tong Cheng, Junrui Li, Brian Shoichet, Yuh Nung Jan, Yifan Cheng, Lily Yeh Jan

**Affiliations:** 1 Department of Physiology, University of California San Francisco (UCSF) School of Medicine, San Francisco, CA USA; 2 Department of Biochemistry and Biophysics, University of California San Francisco (UCSF) School of Medicine, San Francisco, CA USA; 3 Howard Hughes Medical Institute; UCSF, San Francisco, CA USA; 4 BioDuro-Sundia Inc., Irvine, CA USA; 5 Department of Pharmaceutical Chemistry, University of California San Francisco (UCSF) School of Pharmacy, San Francisco, CA USA; 6 Present Address: Dewpoint Therapeutics, Boston, MA USA

**Keywords:** Cryoelectron microscopy, Chloride channels, Permeation and transport, Electrophysiology

## Abstract

The dual functions of TMEM16F as Ca^2+^-activated ion channel and lipid scramblase raise intriguing questions regarding their molecular basis. Intrigued by the ability of the FDA-approved drug niclosamide to inhibit TMEM16F-dependent syncytia formation induced by SARS-CoV-2, we examined cryo-EM structures of TMEM16F with or without bound niclosamide or 1PBC, a known blocker of TMEM16A Ca^2+^-activated Cl^-^ channel. Here, we report evidence for a lipid scrambling pathway along a groove harboring a lipid trail outside the ion permeation pore. This groove contains the binding pocket for niclosamide and 1PBC. Mutations of two residues in this groove specifically affect lipid scrambling. Whereas mutations of some residues in the binding pocket of niclosamide and 1PBC reduce their inhibition of TMEM16F-mediated Ca^2+^ influx and PS exposure, other mutations preferentially affect the ability of niclosamide and/or 1PBC to inhibit TMEM16F-mediated PS exposure, providing further support for separate pathways for ion permeation and lipid scrambling.

## Abstract (web summary)

TMEM16F is a Ca^2+^ activated ion channel and lipid scramblase involved in cell fusion. Here authors determine cryo-EM structures of TMEM16F with or without bound blockers, such as the FDA-approved drug niclosamide.

## Introduction

Linked to the bleeding disorder Scott syndrome^1^, TMEM16F with dual functions of Ca^2+^-activated ion channel^2^ and Ca^2+^-activated lipid scramblase^3,4^ is required not only for PS exposure but also for the release of microvesicles (also known as microparticles) that bud off the cell membrane^2,5^ – two physiological functions critical for blood coagulation^6^. Generation of extracellular vesicles via cell membrane budding is also critical for TMEM16F-dependent bone formation^7^, neutrophil mediated protection of arthritis^8^, and membrane repair^9^. Notably, the ability of TMEM16F to mediate Ca^2+^ influx^2,10–12^ provides positive feedback to enhance the dual functions of TMEM16F that are activated by Ca^2+^. Indeed, both the ion channel function and the lipid scramblase function of TMEM16F are required for extracellular vesicle generation^10^. Just how TMEM16F mediates both ion permeation and lipid translocation between the inner and outer leaflets of the cell membrane remains an intriguing open question.

TMEM16 proteins form dimers^13,14^; each subunit comprises 10 transmembrane helices (TMs) and contains its own ion conduction pore surrounded by TM3-TM7. Ca^2+^-dependent activation involves direct binding of Ca^2+^ ions to two contiguous Ca^2+^-binding sites formed by TM6-TM8. Structural analyses of Ca^2+^-free and Ca^2+^-bound states of the TMEM16A Ca^2+^-activated chloride channel and TMEM16F reveal that TM6 undergoes major Ca^2+^-dependent conformational rearrangements^15–17^. TMEM16F and TMEM16A have similar structures with protein-enclosed pore for ion permeation^4,15–17^.

Recent studies revealing that the protein-enclosed open pore stabilized via activating mutations of TMEM16F spans half the width of a bilayer – in the middle of the membrane^18^ – further reinforce the idea that a separate pathway for lipid translocation across the bilayer is necessary. Lipid scrambling could be facilitated by membrane distortion and thinning that is associated with TMEM16F protein conformations stabilized by the presence of PIP_2_ or activating mutations^17,18^. In support of a separate pathway for lipid scrambling, we have shown that mutations of lipid coordinating residues as well as charged residues in the vicinity of membrane distortion specifically alter the latency of PS exposure but not Ca^2+^ influx following chemical induction for extracellular vesicle generation^17^. It is important to obtain additional structural indications for a separate pathway outside the ion conduction pore, to help us better understand the mechanism for lipid scrambling.

TMEM16F-mediated PS exposure plays a critical role in cell fusion^19,20^ and enveloped virus entry^21,22^. Moreover, TMEM16F modulators can inhibit SARS-CoV-2 virus entry^22^ and syncytia formation triggered by interactions between the Spike viral protein and its ACE2 receptor on adjacent cells^23^. Whereas the FDA-approved drug niclosamide that blocks TMEM16F-dependent syncytia formation is under examination in clinical trials^24,25^ (clinicaltrials.gov) for treatment of severe COVID-19, niclosamide also inhibits TMEM16A and mitigates the symptoms of airway diseases in mice^26,27^. Niclosamide inhibition of TMEM16A may be influenced by the permeant ions^28,29^, thus resembling the action of the TMEM16A pore blocker 1PBC^30^ rather than other TMEM16A inhibitors such as benzbromorone^31^. Thus far, assessments of the effects of niclosamide and 1PBC on TMEM16F concern the current that is carried predominantly by monovalent ions^26,29^. Hence it is important to examine how niclosamide and 1PBC affect the TMEM16F-mediated Ca^2+^ influx and PS exposure, and to characterize their molecular interactions with TMEM16F. Characterization of the TMEM16F modulator binding site is critical for understanding the mechanism of action and for designing drugs that target TMEM16F.

**Fig. 1:**
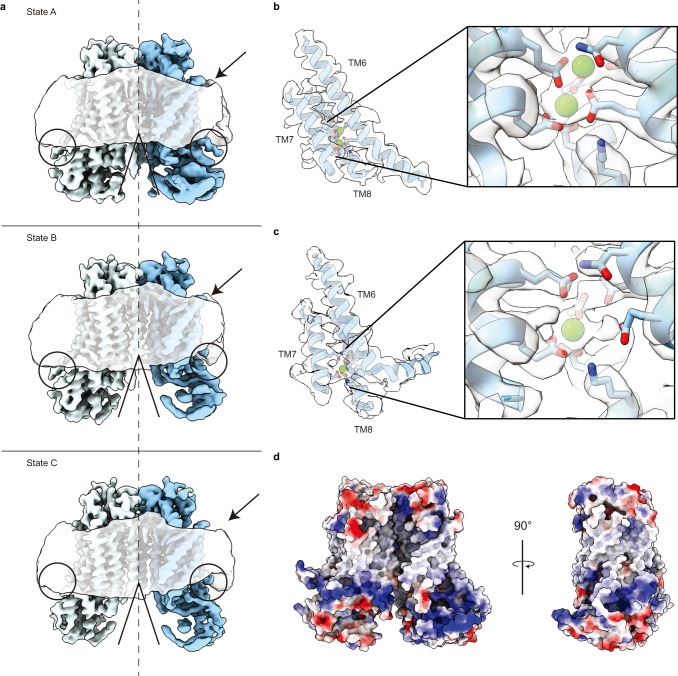
Identification of three distinct states in drug-free TMEM16F. **a** Gaussian filtered density of the nanodisc and unsharpened density of the protein dimer. Unsharpened cryo-EM density and atomic model for three states with different conformations of TM6. Right, Ca^2+^ binding sites, with sharpened cryo-EM density in semitransparent outline and the residues depicted as sticks colored by heteroatom, in (**b**) monomer with extended TM6 and (**c**) monomer with kinked TM6. **d** Electrostatic surface of the asymmetric TMEM16F dimer, where white represents hydrophobic areas and blue and red correspond to positively and negatively charged regions, respectively.

**Fig. 2:**
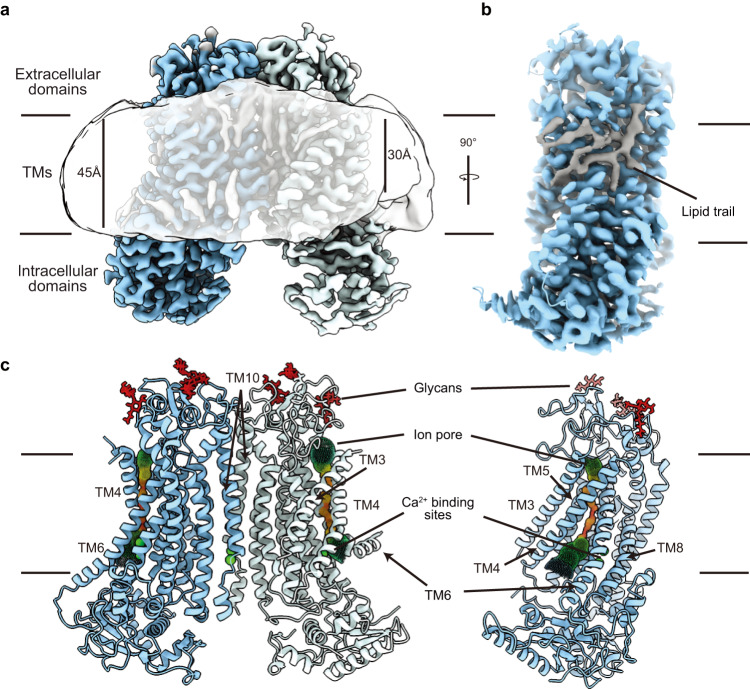
Cryo-EM analysis reveals asymmetry of the TMEM16F dimer. **a** Cryo-EM density of the asymmetric state of the TMEM16F dimer with the monomers colored blue and light blue, respectively, and the lipid densities in grey. The gaussian filtered cryo-EM density (semitransparent) reveals distortion of the lipid nanodisc. **b** Side view of a TMEM16F monomer (blue) highlighting the trail of lipids (grey) covering the TM region. **c** Front and side view of the atomic model of the asymmetric state of TMEM16F with Ca^2+^ atoms and glycans shown in green and red, respectively. The ion conduction channel identified by HOLE is represented by spheres colored in rainbow scale based on the local width of the channel, where red <1.5 Å and blue >7.5 Å.

**Fig. 3:**
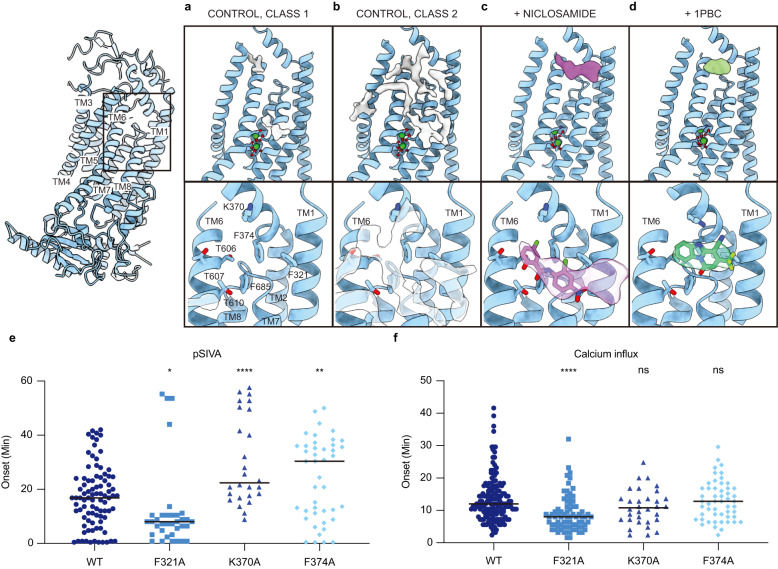
Niclosamide and 1PBC in the same hydrophobic groove of TMEM16F. Atomic model of the TM1-TM6 region of (**a**) Class 1 and (**b**) Class 2 of the drug-free TMEM16F, (**c**) niclosamide-bound TMEM16F and (**d**) 1PBC-bound TMEM16F. In each case, the additional cryo-EM densities found in the area are shown. Below, zoom into the TM1-TM6 groove with the residues shown as sticks and colored by heteroatom and the additional density found within the pocket shown in semitransparent outline. Structures of niclosamide and 1PBC as determined by computational docking using Glide are shown in purple and green, respectively. Bottom panels, differential effects of K370A and F374A mutations on TMEM16F-mediated (**e**) PS exposure (n = 84 for WT; 35 for F321A; 26 for K370A and 43 for F374A) and (**f**) Ca^2+^ influx (n = 162 for WT; 76 for F321A; 32 for K370A and 52 for F374A). At least three independent experiments have been performed for each condition, each with distinct cell populations to assess biological rather than technical variability. The mean ± SEM is shown along with the statistical significance determined by unpaired t-test (two-tailed) for each mutant as compared to the wildtype control (F321A: *p* = 0.0254; K370A: *p* < 0.0001; F374A: *p* = 0.0051 in (**e**) and F321A: *p* < 0.0001; K370A: *p* = 0.1003; F374A: *p* = 0.9862 in (**f**), ^∗^*p* < 0.05; ^∗∗^*p* < 0.01; ^∗∗∗^*p* < 0.001; ^∗∗∗∗^*p* < 0.0001).

**Fig. 4:**
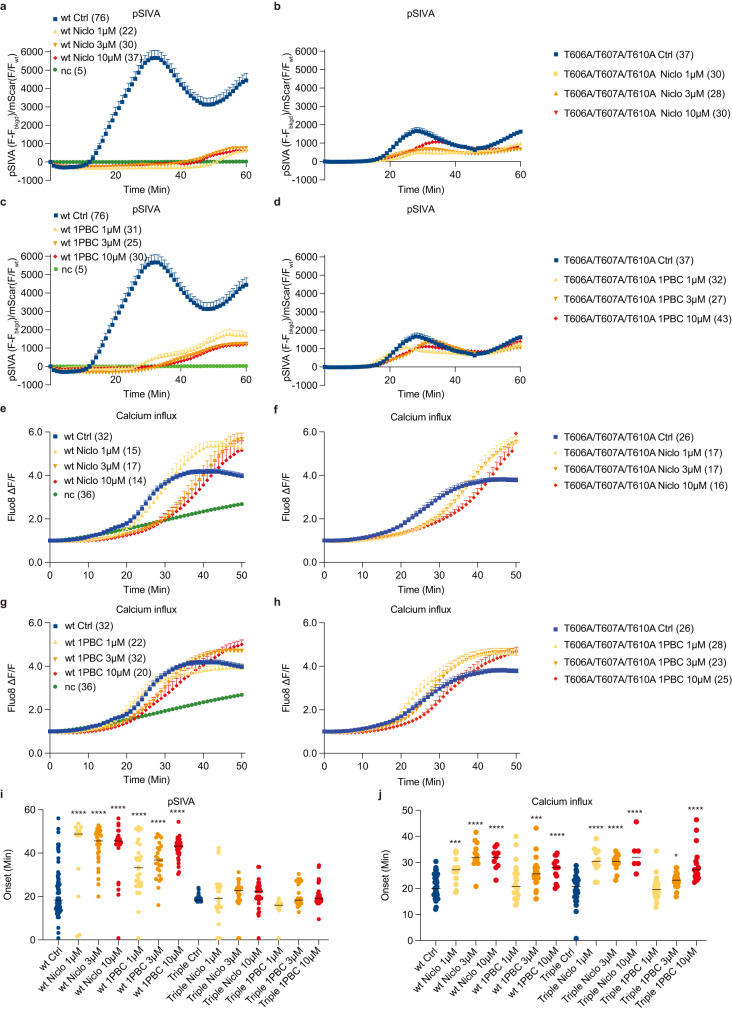
Functional validation of the drug binding site in TMEM16F. Representative curves of live imaging of TMEM16F-dependent PS exposure (**a**–**d**); and Ca^2+^ influx (**e**–**h**). NC is negative control with stable cell line expressing mScarlet rather than TMEM16F tagged with mScarlet. Data are represented as mean ± SEM. Scattered dot plots of time of onset of TMEM16F-dependent PS exposure (**i**) n = 76 for wt Ctrl; 14 for wt Niclo 1 μM; 29 for wt Niclo 3 μM; 25 for wt Niclo 10 μM; 28 for wt 1PBC 1 μM; 25 for wt 1PBC 3 μM; 29 for wt 1PBC 10 μM; n = 23 for Triple Ctrl; 25 for Triple Niclo 1 μM; 22 for Triple Niclo 3μM; 30 for Triple Niclo 10 μM; 28 for Triple 1PBC 1 μM; 25 for Triple 1PBC 3 μM; 42 for Triple 1PBC 10 μM and Ca^2+^ influx (**j**) n = 31 for wt Ctrl; 15 for wt Niclo 1 μM; 12 for wt Niclo 3 μM; 9 for wt Niclo 10 μM; 22 for wt 1PBC 1 μM; 31 for wt 1PBC 3 μM; 15 for wt 1PBC 10 μM; n = 24 for Triple Ctrl; 11 for Triple Niclo 1 μM; 13 for Triple Niclo 3 μM; 6 for Triple Niclo 10 μM; 28 for Triple 1PBC 1 μM; 23 for Triple 1PBC 3 μM; 17 for Triple 1PBC 10 μM. Time of onset could not be determined for time courses with a linear rather than sigmoidal rise. At least three independent experiments have been performed for each condition, each with distinct cell populations to assess biological rather than technical variability. The mean ± SEM is shown along with the statistical significance determined by unpaired t-test (two-tailed) for wildtype or mutant with 1PBC or Niclo as compared to its vehicle control (wt Niclo 1 μM: *p* < 0.0001; wt Niclo 3 μM: *p* < 0.0001; wt Niclo 10 μM: *p* < 0.0001; wt 1PBC 1 μM: *p* < 0.0001; wt 1PBC 3 μM: *p* < 0.0001; wt 1PBC 10 μM: *p* < 0.0001; Triple Niclo 1 μM: *p* = 0.8613; Triple Niclo 3 μM: *p* = 0.3651; Triple Niclo 10 μM: *p* = 0.1865; Triple 1PBC 1 μM: *p* < 0.0001; Triple 1PBC 3 μM: *p* = 0.1166; Triple 1PBC 10 μM: *p* = 0.3885 in (**i**) and wt Niclo 1 μM: *p* = 0.0004; wt Niclo 3 μM: *p* < 0.0001; wt Niclo 10 μM: *p* < 0.0001; wt 1PBC 1 μM: *p* = 0.3469; wt 1PBC 3 μM: *p* = 0.0003; wt 1PBC 10 μM: *p* < 0.0001; Triple Niclo 1 μM: *p* < 0.0001; Triple Niclo 3 μM: *p* < 0.0001; Triple Niclo 10 μM: *p* < 0.0001; Triple 1PBC 1 μM: *p* = 0.8231; Triple 1PBC 3 μM: *p* = 0.0161; Triple 1PBC 10 μM: *p* < 0.0001 (**j**), ∗*p* < 0.05; ∗∗*p* < 0.01; ∗∗∗*p* < 0.001; ∗∗∗∗*p* < 0.0001). Triple: T606A/T607A/T610A.

**Fig. 5:**
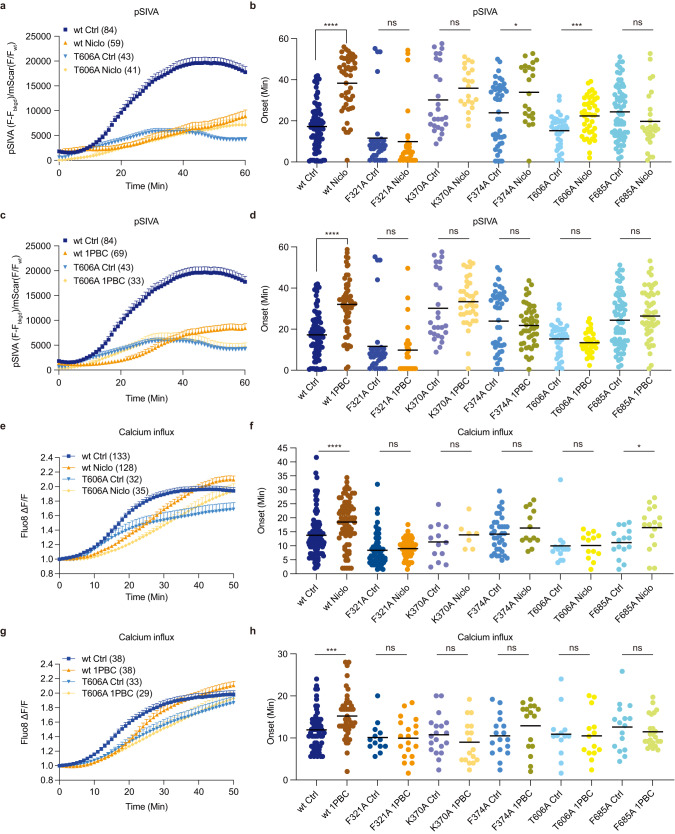
Functional validation of the drug binding site in TMEM16F. Representative curves of live imaging of TMEM16F-dependent PS exposure (**a**) and (**c**); and Ca^2+^ influx (**e**) and (**g**). Data are represented as mean ± SEM. Scattered dot plots of time of onset of TMEM16F-dependent PS exposure [(**b)** and (**d**)] n = 84 for wt Ctrl; 47 for wt Niclo; 58 for wt 1PBC; 35 for F321A Ctrl; 30 for F321A Niclo; 24 for F321A 1PBC; 26 for K370A Ctrl; 21 for K370A Niclo; 38 for K370A 1PBC; 43 for F374A Ctrl; 33 for F374A Niclo; 48 for F374A 1PBC; 39 for T606A Ctrl; 41 for T606A Niclo; 33 for T606A 1PBC; 69 for F685A Ctrl; 27 for F685A Niclo; 50 for F685A 1PBC, and Ca^2+^ influx [(**f**) n = 103 for wt Ctrl; 77 for wt Niclo; 63 for F321A Ctrl; 58 for F321A Niclo; 13 for K370A Ctrl; 7 for K370A Niclo; 35 for F374A Ctrl; 13 for F374A Niclo; 14 for T606A Ctrl; 13 for T606A Niclo; 15 for F685A Ctrl and 16 for F685A Niclo and (**h**) n = 59 for wt Ctrl; 48 for wt 1PBC; 13 for F321A Ctrl; 19 for F321A 1PBC; 19 for K370A Ctrl; 17 for K370A 1PBC; 17 for F374A Ctrl; 16 for F374A 1PBC; 13 for T606A Ctrl; 14 for T606A 1PBC; 16 for F685A Ctrl; 23 for F685A 1PBC] after chemical induction via 25 mM paraformaldehyde (PFA) and 2 mM dithiothreitol (DTT), in the absence (Ctrl) or presence of 3 μM niclosamide (Niclo) or 3 μM 1PBC. Time of onset could not be determined for time courses with a linear rather than sigmoidal rise. At least three independent experiments have been performed for each condition, each with distinct cell populations to assess biological rather than technical variability. The mean ± SEM is shown along with the statistical significance determined by unpaired t-test (two-tailed) for wildtype or each mutant with 1PBC or Niclo as compared to its vehicle control (wt Niclo: *p* < 0.0001; wt 1PBC: *p* < 0.0001; F321A Niclo: *p* = 0.6599; F321A 1PBC: *p* = 0.6372; K370A Niclo: *p* = 0.1682; K370A 1PBC: *p* = 0.3624; F374A Niclo: *p* = 0.0127; F374A 1PBC: *p* = 0.4348; T606A Niclo: *p* = 0.0003; T606A 1PBC: *p* = 0.2603; F685A Niclo: *p* = 0.1268; F685A 1PBC: *p* = 0.1766 in (**b**) and (**d**), wt Niclo: *p* < 0.0001; F321A Niclo: *p* = 0.8285; K370A Niclo: *p* = 0.3827; F374A Niclo: *p* = 0.2960; T606A Niclo: *p* = 0.9689; F685A Niclo: *p* = 0.0300 in (**f**) and wt 1PBC: *p* = 0.0008; F321A 1PBC *p* = 0.9213; K370A 1PBC: *p* = 0.2893; F374A 1PBC: *p* = 0.2048; T606A 1PBC: *p* = 0.8562; F685A 1PBC: *p* = 0.4550 in (**h**), ∗*p* < 0.05; ∗∗*p* < 0.01; ∗∗∗*p* < 0.001; ∗∗∗∗*p* < 0.0001). Niclo: Niclosamide.

In this study, we determined cryo-EM structures of TMEM16F that is bound to Ca^2+^ but not to niclosamide or 1PBC in three distinct states that reveal structural asymmetry, thereby lending further support for the association of membrane distortion and thinning with specific protein conformation (Fig. 1). Notably, some of the TMEM16F proteins had a lipid trail in a groove flanked by TM1 and TM6 outside the ion conduction pore (Fig. 2). We also determined the structures of TMEM16F with niclosamide or 1PBC in a binding pocket near the extracellular end of the groove flanked by TM1 and TM6; their presence eliminated the appearance of a lipid trail. Moreover, alanine substitution of K370 and F374 on TM2 – residues lining the lipid trail – specifically altered TMEM16F-mediated PS exposure but not Ca^2+^ influx (Fig. 3). Both niclosamide and 1PBC exhibited dose-dependent inhibition of TMEM16F-mediated Ca^2+^ influx and PS exposure, while alanine substitutions of three threonines in the binding pocket abolished the ability of niclosamide and 1PBC to inhibit PS exposure but not Ca^2+^ influx (Fig. 4). Taken together with the finding that alanine substitution of several residues in the binding pocket preferentially affects the ability of niclosamide or 1PBC to inhibit TMEM16F-mediated PS exposure (Fig. 5), our study provides evidence for a pathway for lipid scrambling outside the ion conduction pore.

## Results

### Cryo-EM analysis reveals an asymmetric state of the TMEM16F dimer

The presence of phosphatidylinositol 4,5-bisphosphate (PIP_2_) is important for activation of TMEM16F^32,33^. Combination of lipid nanodisc technology with single particle cryo-EM allows structural analysis of membrane proteins embedded in a lipid bilayer^34,35^, which is critical for TMEM16 proteins and other membrane proteins that are modulated by lipids. However, TMEM16 proteins in nanodiscs present strong preferred orientation in particle distribution, severely limiting the attainable resolution of cryo-EM structures of TMEM16 proteins and hampering the study of these proteins in the context of a lipid bilayer^15,17^. We overcame this limitation by collecting data from tilted specimen and implementing an image processing pipeline that allowed us to systematically determine sub 3.5 Å structures of TMEM16F in lipid nanodiscs in the presence or absence of different modulators (see “Methods”) (Supplementary Figs. 1, 2, 3 and Supplementary Table 1).

First, we determined multiple structures of TMEM16F in the presence of Ca^2+^ and PIP_2_. These structures represent different conformations of TMEM16F without bound niclosamide or 1PBC. The quality of these reconstructions enables atomic model building of the TM helices, most of the extracellular and intracellular domains, as well as Ca^2+^ ions and dozens of lipid densities associated with the protein (Figs. 1, 2 and Supplementary Table 1). The resolution achieved in our structural analyses has made it possible to detect glycans on the extracellular loops as well as the channel pore for ion permeation and the Ca^2+^ binding sites (Fig. 2c). We did not impose C2 symmetry and identified 3 distinct states (Fig. 1a) with major differences in the conformation of TM6 and the number of Ca^2+^ atoms bound in each monomer. In State A, both monomers are bound to 2 Ca^2+^ ions and present a clear density for an extended TM6 (Fig. 1a, b). In State B, one monomer has 2 Ca^2+^ ions and a straight TM6, whereas the other monomer appears to contain a single Ca^2+^ ion, as density for the second Ca^2+^ ion is significantly weaker (Fig. 1a, c, d). In this single Ca^2+^-bound monomer, TM6 presents a kink at P628 (Fig. 2c). Thus, this structure represents an asymmetric state of the dimer (Fig. 2). In State C, both monomers contain only 1 Ca^2+^ ion and bent TM6 (Fig. 1a). Comparison between these 3 classes reveals that straightening of TM6 correlates with binding of the second Ca^2+^ ion, whereas kinking of TM6 is associated with an outward rigid body motion of the intracellular domain that brings it closer to the nanodisc (Figs. 1a and 2a). Moreover, bending of TM6 directly correlates with distortion of the nanodisc and significant thinning of the membrane at the kinking position (Figs. 1a and 2a). Consistent with our previous study of TMEM16F^17^, these observations support the notion that kinking of TM6 at P628 causes membrane distortion.

Our reconstructions also reveal some interesting features (Supplementary Fig. 4), including glycans and conserved disulfide bonds in the extracellular region (Supplementary Fig. 4a, b), as well as the presence of a third Ca^2+^ ion coordinated by E395 on TM2 as well as S854 and D859 on TM10, near the dimer interface in the intracellular region of the protein (Supplementary Fig. 4c). These features are likely present in previous reconstructions but not detected due to limited resolution. In fact, a similar Ca^2+^-binding site has been found in TMEM16F^4^ and TMEM16K^36^, and recent studies indicate that an equivalent third Ca^2+^-binding site in TMEM16A allosterically regulates channel activity^37^.

We are also able to unambiguously assign the residues of TM4 and precisely determine the pore-lining residues on TM4 (Supplementary Fig. 4d). These residues form a network of OH-containing side chains along the hydrophilic pore that constitutes an ideal environment for ion conduction across the membrane (Fig. 2c). However, the ion conduction pore is closed in all states resolved in this study and its hydrophilic interior is not accessible to lipids from the surrounding membrane (Supplementary Fig. 4e).

### TM1 and TM6 form a hydrophobic groove that can be occupied by lipids

In all three classes, we noticed a trail of densities that appear to correspond to a mixture of multiple lipids extending across the entire lipid bilayer along the membrane-facing surface of the TMEM16F monomer (Fig. 2b). A hydrophobic groove formed between TM1 and TM6 near the extracellular edge of the membrane appears to play a major role in accommodating these lipids. Intriguingly, this area corresponds to the position where membrane thinning occurs (Fig. 2a). To further investigate these lipid densities, we combined particles from all three States and carried out focused classification around this groove in a single monomer (See “Methods”) (Fig. 3 and Supplementary Fig. 1). The particles clustered primarily to 2 classes that each contained approximately 40% of the particles and rendered 3.1 Å resolution structures (Supplementary Figs. 1 and 2). The overall protein organization of Class 1 and 2 is essentially indistinguishable (Fig. 3a, b). However, Class 1 almost entirely lacks lipid densities in the TM1-TM6 groove (Fig. 3a), whereas Class 2 has strong density for numerous lipids in this area (Fig. 3b). This indicates that our dataset contains a mixture of monomers in lipid-free and lipid-bound states, raising the intriguing possibility that the lipid trail aligns with the pathway for lipid scrambling.

To test this possibility, we evaluated mutations of residues adjacent to the lipid trail via live imaging, by employing the protocol of chemical induction of giant plasma membrane vesicle formation that involves TMEM16F-mediated Ca^2+^ influx as well as TMEM16F-mediated PS exposure in HEK293 cells^10,17^. Whereas we conducted live imaging for 50 min after chemical induction, we focused our analyses on the latency for onset of Ca^2+^ influx and PS exposure, to avoid complications arising from downstream cellular processes. Notably, alanine substitution of K370 and F374 on the TM1-TM2 loop significantly lengthened the latency for PS exposure, without affecting the latency for Ca^2+^ influx. Compared to the wildtype control, F321A shortened the onset latency of Ca^2+^ influx by nearly twofold (Fig. 3f) and reduced the onset latency of the PS exposure from 17.23 min to 11.61 min (Fig. 3e), while K370A significantly delayed the onset of PS exposure to 30.16 min (Fig. 3e) without affecting the onset latency of Ca^2+^ influx (Fig. 3f), as did F374A. The specific involvement of these two residues lining the lipid trail on lipid scrambling supports the notion that the lipid trail could correspond to the lipid scrambling pathway.

### Niclosamide binding in the hydrophobic groove formed between TM1 and TM6

Niclosamide is an FDA-approved drug that has recently emerged as a promising drug for treating severe cases of COVID-19^24,25^ (clinicaltrials.gov), and its propensity to inhibit syncytia formation has been attributed to its ability to inhibit TMEM16F^23^. Seeking to determine the binding site of this antagonist, we added 50 μM niclosamide to our biochemical preparation and imaged this sample following identical image processing pipeline as in the dataset presented above (Supplementary Figs. 1, 3). In this case, however, focused classification around the TM1-TM6 groove rendered 3 classes. Like in our control sample, Classes 1 and 2 are distinguished by the absence or presence of lipids in the groove. Class 3 contains a well-defined density in the TM1-TM6 groove that fits niclosamide well while no trail of lipid densities is found in the hydrophobic pocket (Fig. 3c). The niclosamide-like density contacts F321 on TM1, K370 on the TM1-TM2 loop, T606, T607 and T610 on TM6, and F685 and L687 on the TM7-TM8 loop (Fig. 3c). The resolution of our reconstruction is insufficient to unambiguously determine the precise pose of the molecule within the density. To gain some insight into how niclosamide may be oriented within TMEM16F, the compound was computationally docked using the Glide docking software. Using only the atomic model of TMEM16F (without access to our cryo-EM density map or pre-assigning any residues for drug interaction), the software identified this pocket as the most likely binding site and the highest-ranking pose fits our cryo-EM density well (Fig. 3c and Supplementary Fig. 5). Notably, this pose had the lowest binding energy. Taken together, our structural and computational data show that niclosamide binds TMEM16F at the hydrophobic groove formed between TM1 and TM6 and that binding of niclosamide prevents lipids from occupying this pocket.

### 1PBC and niclosamide target the same binding site in TMEM16F

To elucidate the binding site of 1PBC, we supplemented our TMEM16F sample with 100 μM 1PBC. Here too we identified 3 distinct classes that closely resemble the 3 states observed in our drug-free sample. However, lipid densities along the membrane-facing surface of each monomer are absent. Instead, in all three classes we found a strong oval-shaped density in the same hydrophobic groove identified as the drug binding site in our niclosamide-supplemented dataset (Fig. 3d). This density, which is remarkably different from the lipid-like and niclosamide-like densities in our drug-free and niclosamide-bound structures, fits 1PBC well. Overlay of the 1PBC-bound structure with our control revealed subtle side chain rearrangements of the residues surrounding this density (Supplementary Fig. 4f). Computational docking using Glide identified a pose for 1PBC that fits our density map well (Fig. 3d and Supplementary Fig. 5).

We further performed computation docking into TMEM16F with alanine substitutions of residues in the binding pocket. We found higher binding energies of niclosamide and 1PBC for the mutant TMEM16F with F321A or T610A mutation (Supplementary Fig. 5c, e), without identifying poses that match the cryo-EM density. Thus, our data show that 1PBC and niclosamide target the same site in TMEM16F and appear to replace bound lipids in the hydrophobic groove formed between TM1 and TM6 (Fig. 3).

### Niclosamide and 1PBC inhibit TMEM16F-mediated Ca^2+^ influx and PS exposure

Niclosamide is known to inhibit both TMEM16F and TMEM16A channels^26^. Given the structural similarities between both paralogs, we reasoned that 1PBC, a potent inhibitor of TMEM16A, might also modulate TMEM16F. First, we explored whether TMEM16F-mediated lipid scrambling is inhibited by 1PBC and niclosamide, by imaging PS exposure using pSIVA, a fluorescent annexin derivative (Fig. 4a, c). Upon chemical induction to induce giant plasma membrane vesicle formation^10^, application of 1 μM, 3 μM or 10 μM 1PBC or niclosamide delayed the onset for PS exposure (Fig. 4i). The average onset for PS exposure in vehicle controls was 17.23 min. 1PBC or niclosamide at all three concentrations robustly delayed the onset of TMEM16F-mediated PS exposure by ~2-fold (Fig. 4i). Next, we tested for the ability of niclosamide and 1PBC to inhibit TMEM16F-mediated Ca^2+^ influx, and measured Ca^2+^ influx using Fluo8 as a small molecule Ca^2+^ reporter dye (Fig. 4e, g). Application of 3 μM or 10 μM, but not 1 μM, of 1PBC led to a significant delay in the onset of TMEM16F-mediated Ca^2+^ influx upon chemical induction^10,17^, as did application of niclosamide at 1 μM, 3 μM and 10 μM (Fig. 4j). Thus, niclosamide and 1PBC potently inhibits TMEM16F function by reducing both Ca^2+^ influx and lipid scrambling activity.

### Differential effects of niclosamide and 1PBC on the Ca^2+^ influx and PS exposure mediated by wildtype and mutant TMEM16F

We further tested the effect of 1 μM, 3 μM and 10 μM niclosamide and 1PBC on the triple mutant with alanine substitutions of T606, T607 and T610 on PS exposure (Fig. 4b, d) and Ca^2+^ influx (Fig. 4f, h). Our finding that 1PBC inhibited PS exposure to a greater extent than Ca^2+^ influx is reminiscent of the differential effects of recently identified TMEM16F modulators^22^. The triple mutation T606A/T607A/T610A drastically reduced the ability of niclosamide and 1PBC to inhibit PS exposure (Fig. 4i) while having mild effect on 1PBC inhibition of Ca^2+^ influx (Fig. 4j). It thus appears that interaction of niclosamide or 1PBC with some residues in this binding pocket has a greater impact on the lipid scrambling pathway than the ion permeation pathway.

### Functional validation of the drug binding site in TMEM16F

Similar to mutagenesis studies mentioned above, we generated stable cell lines expressing wildtype or mutant TMEM16F-mScarlet containing alanine substitutions of the residues surrounding the inhibitor densities: F321 on TM1, K370 and F374 on the TM1-TM2 loop, T606 on TM6, and F685 on the TM7-TM8 loop. In wild type controls, both 1PBC and niclosamide significantly delayed the onset of internal Ca^2+^ rise and PS exposure (Fig. 5 and Supplementary Fig. 6). The inhibitory effect of both antagonists was significantly decreased by all the mutations, confirming that these residues are important for binding these inhibitors (Fig. 5). In fact, the F321A and K370A mutations almost completely obliterated the inhibitory effects on the onset of both Ca^2+^ rise and PS exposure (Fig. 5).

We relied on measurements of TMEM16F-mediated Ca^2+^ influx and PS exposure upon chemical induction of giant plasma membrane vesicle formation^10,17^ for testing the effects of niclosamide and 1PBC, because whole-cell currents of TMEM16F expressing cells require several minutes to activate^12^, and application of 1 μM or 3 μM niclosamide led to an increase in whole-cell current that differed from TMEM16F currents based on reversal potential measurements, in control cells as well as cells with heterologous expression of TMEM16F (Supplementary Fig. 7a, b). This finding is reminiscent of a recent report of niclosamide induced activation of a non-TMEM16A conductance endogenous to HEK293 cells^27^. Moreover, the Q559K mutation alters the ion selectivity of TMEM16F currents recorded from inside-out patches^2,4,11,12,38^ but not the whole-cell currents of TMEM16F expressing cells^12^. The inability of TMEM16F mutations to alter whole-cell currents, which take several minutes to develop, raises the possibility that cellular processes downstream of TMEM16F activation contribute to the whole-cell current. Whereas it is conceivable that niclosamide or 1PBC applied to the inside-out membrane patch may be able to reach their binding pocket, the rapid desensitization of TMEM16F currents^11,32^ precluded reliable assessments of drug effects via comparisons with control recordings after washout (Supplementary Fig. 7). We found that the F321A mutation that affected both Ca^2+^ influx and PS exposure, but not the K370A and F374A mutations with specific effect on PS exposure (Fig. 3e, f), enhanced the Ca^2+^ sensitivity of TMEM16F currents recorded from inside-out patches (Supplementary Fig. 8a) We also tested for the ability of the TMEM16A inhibitor benzbromarone^31^ to affect TMEM16F-mediated Ca^2+^ influx and PS exposure (Supplementary Fig. 8b, c). Whereas treatment with 0.3 μM benzbromarone increased the latency for onset of both Ca^2+^ influx and PS exposure following chemical induction, its effects at higher concentrations were less clearly, possibly owing to off-target effects^27^.

For a structural assessment of the impact of niclosamide and 1PBC on the TMEM16F channel pore for ion permeation, we used HOLE to measure the pore diameter of TMEM16F associated with niclosamide and 1PBC (Supplementary Fig. 9). The diameter of the pore at the constrictions is less than 1 Å, with the monomer that is more stably associated with niclosamide or 1PBC displaying a more constricted pore (Supplementary Fig. 9c, f). This suggests that the binding of niclosamide and 1PBC may further collapse the pore.

In summary, we show that residues in the TM1–TM6 groove are important for niclosamide- and 1PBC-mediated inhibition of TMEM16F and this area is critical for scramblase activity, providing further evidence for a separate pathway for lipid scrambling outside the ion conduction pore.

## Discussion

**Fig. 6:**
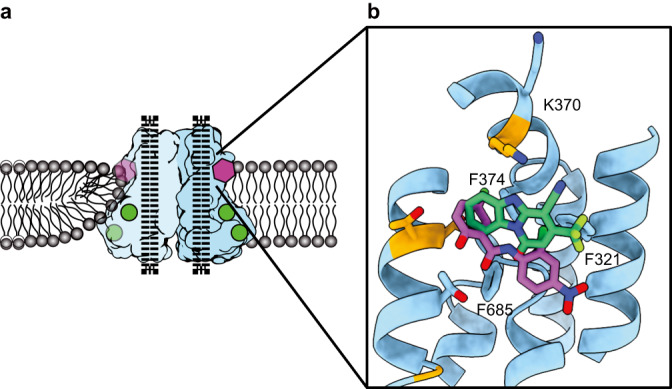
The drug binding pocket identified in TMEM16F. **a** Schematic representation of the TMEM16F dimer (light blue and blue) embedded in a lipid bilayer (grey), where Ca^2+^ atoms are shown as green circles and the inhibitors as a purple polygon and dotted black lines represent the closed ion conduction pore. **b** Structure of the drug binding pocket in TMEM16F with the side chains of the surrounding residues shown as sticks and the non-conserved residues highlighted in orange. Computationally docked structures of niclosamide and 1PBC are shown in purple and green, respectively. All atoms are colored by heteroatom.

In this study we provide several lines of evidence in support of separate pathways for ion permeation and lipid scrambling. First, structural analysis of TMEM16F in lipid nanodiscs supplemented with PIP_2_ reveals 3 distinct states and a direct correlation between kinking of TM6 and membrane distortion (Fig. 1). A continuous trail of lipids connects the intra- and extracellular sides of TMEM16F at the membrane distortion site (Fig. 2a, b). These findings are consistent with our previous structural and mutagenesis data^17^ and support a model for TMEM16F-mediated scrambling of lipids, whereby TMEM16F distorts the membrane, minimizing the distance between the inner and outer leaflets of the lipid bilayer (Fig. 6). We further find that residues along this lipid trail, such as K370 and F374 on the TM1–TM2 loop, are important for scramblase activity. In fact, K370 is a positively charged residue that is ideally positioned for interacting with negatively charged phospholipid headgroups at the membrane interface. The fact that TMEM16A, which cannot scramble lipids, contains an alanine in this position reinforces the notion that this basic residue is critical for lipid scrambling. Together, our data suggest that the lipid trail we identify on TMEM16F might correspond to the pathway for lipid scrambling. We propose that the lipids “surf” along this membrane-facing groove, crossing between the inner and outer leaflets through a path that does not directly involve the hydrophilic ion conduction pore.

Secondly, we found that the potency for 1PBC inhibition of TMEM16F-mediated PS exposure (Fig. 4e) is greater than the potency for its inhibition of TMEM16F-mediated Ca^2+^ influx (Fig. 4f). Moreover, the triple mutation T606A/T607A/T610A of TMEM16F abolished the ability of niclosamide and 1PBC to inhibit PS exposure (Fig. 4e) but not Ca^2+^ influx (Fig. 4f). It thus appears that interactions of niclosamide and 1PBC with these residues in the binding pocket likely have differential effects on the scramblase activity of TMEM16F. Taken together with the dependence of PS exposure on residues on the TM1-TM2 loop that are not pore-lining helices but are lining the lipid trail (Fig. 3e, f), support the notion of a separate pathway for lipid scrambling outside of the ion conduction pore.

Our findings of separate pathways for ion permeation and lipid scrambling are consistent with recent studies of TMEM16F as well as fungal TMEM16 homologs. Whereas the credit card model has been proposed for lipid scramblases such as nhTMEM16^13,14,39^, recent studies of afTMEM16 reveal pronounced membrane thinning resulting from rearrangement of lipids in the intracellular vestibule, leading to the conclusion that an open hydrophilic pathway is not required for lipid scrambling^40^. Recent studies of constitutively active TMEM16F mutants further reveal an open pore being surrounded by pore-lining helices next to membrane distortion that may facilitate lipid scrambling, leading to the conclusion that lipid translocation across the membrane likely takes place outside of the pore^18^. Separate pathways for ions and lipids would allow an ion channel moonlighting as a lipid scramblase flexibility for physiological regulations and pharmacological modulations.

TMEM16 proteins have emerged as important pharmacological targets for the treatment of cancer, asthma and more recently COVID-19^22–25,41^ (clinicaltrials.gov). Our data indicate that niclosamide and 1PBC bind the same binding pocket in TMEM16F (Fig. 6 and Supplementary Fig. 5). With both modulators directly contacting the extracellular-proximal end of TM6, our mutagenesis studies suggest that residues on this helix are critical for drug binding (Fig. 5). TM6 is the main gating element of the channel as well as part of the ion conduction pore in both TMEM16A and TMEM16F^13,14^. We thus speculate that, by binding to the upper part of TM6, these modulators may simultaneously lock the ion conduction pore as well as the gating element in a closed configuration.

Interestingly, the binding site of 1PBC and niclosamide coincides with the position where we observe the maximum degree of membrane distortion and thinning in the TMEM16F structures. In our model for TMEM16F activity, this site corresponds precisely with the entry and exit point of the lipids as they transition between the inner and outer leaflets of the plasma membrane (Figs. 3 and 6). Our structures show that both modulators replace the lipids found in this pocket in our drug-free sample (Fig. 3). This suggests that 1PBC and niclosamide might directly inhibit TMEM16F scramblase activity by physically occluding the path of the lipids across the membrane. In fact, lipid densities along this path are significantly reduced in our 1PBC- and niclosamide-bound TMEM16F structures (Fig. 3). Consistent with a critical role of this region for lipid scrambling, alanine substitutions of residues within the drug binding pocket significantly alter the lipid scrambling activity of TMEM16F in the absence of inhibitors (Fig. 3e).

Like 1PBC and niclosamide, many drug molecules are not specific for any particular TMEM16 paralog and instead broadly target TMEM16 family members^22,27,42^. Extensive efforts have been devoted to identifying TMEM16A modulators^43^. The pore blocker 1PBC, with its TMEM16A inhibition dependent on membrane potential and permeant ion concentrations^30^, has been recently found to associate with the outer pore^29^. Another pore blocker of TMEM16A, A9C, appears to enter the open channel following outer pore rearrangement induced by Ca^2+^ binding, to block ion permeation. A9C may then cause allosteric channel activation possibly involving further conformation changes^44^. It thus appears likely that a TMEM16A modulator may interact with the channel in more than one conformation. The ability to computationally dock 1PBC and niclosamide into a TMEM16A binding site equivalent to that found in TMEM16F^45^, along with the ability of TMEM16A mutations in this region to affect TMEM16A inhibition by niclosamide and 1PBC^45^, could be an indication of multiple binding sites for these TMEM16A modulators, though we cannot exclude the possibility of allosteric effects of the TMEM16A mutations.

Structural analyses of TMEM16F interactions with modulators are important for basic research as well as drug development. Notably, SARS-CoV-2 S induces internal Ca^2+^ rise and TMEM16F activation for PS exposure that facilitates host cell membrane fusion with the viral envelope; inhibitors that reduce TMEM16F lipid scramblase activity exhibit antiviral activities against SARS-CoV-2 infection, while their binding sites remain to be determined^22^. Niclosamide, which blocks SARS-CoV-2-induced syncytia formation and virus replication by inhibiting TMEM16F^23^, is a highly hydrophobic molecule that presents extremely poor solubility in aqueous solutions^46^. Additionally, our whole cell patch clamp electrophysiology experiments (Supplementary Fig. 7) as well as previous studies^27,47^ reveal that niclosamide may activate an ion channel. The identification of hydrophilic residues within the drug binding pocket in TMEM16F that are not conserved in other family members such as TMEM16A opens the door for the development of niclosamide analogs with better pharmacological properties that exclusively target TMEM16F for the treatment of severe COVID-19. Taken together, our work provides evidence for separate pathways in TMEM16F for ion permeation and lipid scrambling as well as a structural framework for designing more potent and more specific modulators against individual members of the TMEM16 family.

## Methods

### Protein expression and purification

TMEM16F was expressed and prepared as previously described^17^ with some modifications. Briefly, the protein was expressed in HEK293s cells, extracted and purified in the presence of DDM/CHS and reconstituted in MSP2N2-SoyPC nanodiscs at a 1:4:100 molar ratio of TMEM16 monomer:MSP:lipid. PIP_2_ was added to the sample after nanodisc reconstitution at a 4:1 molar ratio of PIP_2_:TMEM16 monomer and incubated for 15 min before FPLC. 1-oxo-3-(trifluoromethyl)-1,5-dihydropyrido[1,2-a]benzimidazole-4-carbonitrile (1PBC) was purchased from VITAS-M laboratory (Champaign, IL, USA) and niclosamide was purchased from Sigma Aldrich (St. Louis, MO, USA). 50 mM and 25 mM stocks of 1PBC and niclosamide were freshly prepared in DMSO and EtOH, respectively, and added to the sample to a final concentration of 100 and 50 µM, respectively. The inhibitors and 4 mM CaCl_2_ were added to the sample immediately prior to grid preparation.

### Sample preparation for electron microscopy

For cryo-EM structure determination, 3.5 µl of the sample at approx. 0.7 mg/ml were applied to 300 mesh UltrAuFoil Holey Gold Films R1.2/1.3 (Quantifoil) that had been previously plasma cleaned. The grids were loaded into a Vitrobot (ThermoFisher) with an environment chamber at a temperature of 4 °C and 100% humidity, blotted for 4 s at 0 blotting force with Whatman No.4 filter paper and plunged into a liquid ethane slurry.

### Electron microscopy data acquisition

Cryo-EM data were collected at Janelia HHMI Cryo-EM facility on a ThermoFisher Krios transmission electron microscope (TEM) operating at 300 keV with a Quantum energy filter (Gatan) set to a slit width of 20 eV. Dose-fractionated movies were collected using a Gatan K3 Summit direct electron detector operating in CDS super resolution mode. Collection dose rate was 9 e^−^/pixel/s and the total cumulative dose was 66 e^−^/Å^2^ over 120 frames. Micrographs were collected with a 3 × 3 image shift collection strategy at ×105,000 magnification (0.42 Å/pixel at the specimen level in super resolution mode), with a nominal defocus range of −0.8 to −2.2 μm using semi-automated scripts in SerialEM^48^. All samples presented severe preferred orientation. To address this issue, images were collected at 0°, 30°, 35° and 45° degrees tilt^49^ as specified in Supplementary Table 1.

### Image processing

For each dataset, micrograph frames were aligned using MotionCorr2^50^ and cryoSPARC 3.0^51^ was used for all initial processing: CTF was estimated with CTF Patch and only micrographs with resolution estimations higher than 3.5 Å with confidence values above 97% were further processed. Particles were picked using BlobPicker and extracted with an unbinned box size of 256 pixels. 2D classification was used to eliminate obvious mispicks and selected particles were reconstructed using non-uniform refinement using our previous cryoEM reconstruction of TMEM16F (EMD-20246), respectively, low pass filtered to 30 Å as an initial model. Tilted and untilted datasets were merged and subjected to heterogenous refinement using 3 classes. Of note, as initial models for the heterogenous refinement, we inputted 2 volumes representing of our previous reconstructions, whereas the initial model for class 3 was a volume obtained from a failed ab initio reconstruction. The latter captured empty nanodiscs and damaged particles, allowing us to classify those out. Successive rounds of this processing pipeline rendered high resolution reconstructions that were further processed using local refinement in cryoSPARC. For analysis of the drug binding site in TMEM16F, we performed focused classification without alignment in Relion 3.1. with a Tau value of 20 using a mask around the ligand-binding area. The outputs were subjected to a manual refinement in cisTEM.

### Atomic model building and refinement

Previous structures of TMEM16F (PDB: 6P48) were used as a starting model. The structure was adjusted and novel areas were built de novo using the COOT software package^52^, and further refined using ISOLDE and real-space refinement from the PHENIX package^53^. Chimera X was used for visualization and figures.

### Lipid scrambling assay

Lipid scrambling was assayed as previously reported^10,17^ with some modifications. Briefly, Stable HEK293 cell lines expressing wild-type or mutant mTMEM16F were plated on glass bottom dishes in Opti-MEM medium overnight prior to imaging. Cells were washed twice in HEPES-buffered modified Tyrode’s buffer (10 mM HEPES pH7.5, 150 mM NaCl, 10 mM glucose, 2 mM CaCl_2_) and incubated in the same buffer containing 25 mM paraformaldehyde (PFA)/and 1:100 pSIVA (BioRad, Hercules, CA) for 5 min. Image acquisition began once dithiothreitol (DTT) was added to a final concentration of 2 mM and terminated after 1 h. Image analysis was carried out on each individual cell using the Nikon Elements Software^10^. To calculate the values for time of onset, we fit the pSIVA imaging curve with the Weibull growth model using Graph Pad Prism 9: Y = YM − (YM − Y0)∗exp(−1∗(k∗x)g). The time of onset is the maximum of the second derivative.

### Ca^2+^ rise measurement

Cultured cells were incubated with 1 μM of the calcium reporter dye Fluo-8 AM (AAT Bioquest) for 15 min, washed twice in Dulbecco’s phosphate buffered saline (DPBS), and treated with 25 mM PFA/2 mM DTT in HEPES-buffered modified Tyrode’s buffer (10 mM HEPES pH7.5, 150 mM NaCl, 10 mM glucose, 2 mM CaCl_2_) for live cell imaging on a Nikon-TE2000 inverted microscope (Nikon Instruments, Melville, NY, USA) equipped with a thermostat chamber. Images for both brightfield and Fluo-8 were acquired in parallel once every minute starting 5 min post-treatment for 50 min for a total of 55 min treatment time. To calculate the values for time of onset, we fit the F/F0 curve with the Weibull growth model using Graph Pad Prism 9: Y = YM − (YM − Y0)∗exp(−1∗(k∗x)g). The time of onset is the maximum of the second derivative.

### Electrophysiology

Cells were seeded in a 12-well plate for whole-cell patch clamp recordings. The following day, transfection was carried out by using the Lipofectamine^TM^ 3000 Transfection Reagent (Invitrogen, USA) according to the manufacturer’s instructions. We used TMEM16F-EGFP construct for the drug test recordings. All experiments were performed 20–30 h after transfection. The stable cell lines including wt, F321A, K370A and K374A were used for the calcium sensitivity experiments. The cells were transferred onto a small chamber on the stage of an inverted microscope (TE2000, Nikon, Japan) and attached to coverslip in the small chamber for 10 min prior for the patch recordings. Experiments were performed at room temperature (22–24 °C). The recording chamber was continuously perfused at a flow rate of 1–2 ml/min. Borosilicate patch pipettes (Warner instruments, USA) were pulled from a PC-100 puller (Narishige, Japan) and used to obtain the stable gigaohm seal formation. The pipette resistance was 3–5 MΩ for whole cell recordings, and 2–4 MΩ for inside-out patch configuration. The currents were recorded using an Axopatch 200B patch-clamp amplifier (Molecular Devices, USA). pClamp software v11.2 and Digidata 1440B (Molecular Devices) were used for data acquisition and application of the pulses. Low-pass Bessel filter with 5 kHz cut-off frequency was selected for recordings. Pipette capacitance cancellation up to 90% was achieved in cell-attached modes of every recording. The data were analyzed using pCLAMP software v11.2, OriginPro 8 (OriginLab, USA). For the whole cell recordings, the cells were recorded with the ramp pulse protocol from −100 to +100 mV for 1 s every 10 s followed by holding at 0 mV. The inside out recordings were performed by holding the excised patch at +60 mV. To identify the changes in current–voltage relationship after drug treatment, the step pulse protocol from −100 to +100 mV for 1 s with 20 mV increment every 2 s followed by holding at 0 mV was used.

For whole-cell recordings of the TMEM16F, the bath solution being perfused constantly contained 145 mM NaCl, 10 mM HEPES, 2 mM MgCl_2_, 1 mM CaCl_2_, 10 mM glucose, pH 7.2 with NaOH. The patch pipette solution contained 150 mM NaCl, 10 HEPES, 0.1 mM CaCl_2_, pH 7.2 with NaOH. For inside-out recordings, the bath solution composition for the whole cell recording was used for the composition of the patch pipette solution. The membrane patch was excised to form inside-out configuration in Ca^2+^-free solution: 140 mM NaCl, 10 mM HEPES, 5 mM EGTA, pH 7.2 with NaOH. The recording protocols were chosen so that the reversal potential is near 0 mV. A solution containing 40 μM free calcium was used to activate TMEM16F: 140 mM NaCl, 10 mM HEPES, 0.04 mM CaCl_2_, pH 7.2 with NaOH.

### Docking

We used Glide (Schrödinger, Inc.)^54^ in the docking study. The protein was prepared with a default protocol from Protein Preparation Wizard and OPLS3 force field^55^. We applied Epik^56^ method in the ligand preparation for both 1PBC and niclosamide with the range of pH from 6.4 to 8.4. Standard precision (SP) and extra precision (XP)^57^ docking protocols were used to generate binding poses for both compounds within a 30 × 30 × 30 Å box that encompasses most of the TM region. Top ranking positions within the box were determined based on favorable hydrogen binding interactions and hydrophobic forces.

### Reporting summary

Further information on research design is available in the Nature Portfolio Reporting Summary linked to this article.

### Supplementary information

Supplementary Information

Reporting Summary

### Source data

Source Data

## Data Availability

The data that support this study are available from the corresponding authors upon request. Cryo-EM maps have been deposited in the Electron Microscopy Data Bank (EMDB) under accession codes EMD-41134 (TMEM16F State B), EMD-41137 (TMEM16F, Class I), EMD-41136 (TMEM16F, Class II), EMD-40776 (TMEM16F Niclosamide) and EMD-40768 (TMEM16F, 1PBC). Coordinates have been deposited in the Protein Data Bank (PDB) under accession codes 8TAG (TMEM16F, State B), 8TAL (TMEM16F, Class I), 8TAI (TMEM16F, Class II), 8SUR (TMEM16F, Niclosamide) and 8SUN (TMEM16F, 1PBC). Source data are provided with this paper.
