# Supplementary material for: Identification of a drug binding pocket in TMEM16F calcium-activated ion channel and lipid scramblase
Source: Nat Commun. 2023 Aug 12;14:4874. doi: 10.1038/s41467-023-40410-x (PMC10423226; doi:10.1038/s41467-023-40410-x)
Supplement: Supplementary file 1 — Supplementary Information [file 41467_2023_40410_MOESM1_ESM.pdf]

**Supplementary Table 1. Cryo-EM data collection and refinement statistics**

| Sample | TMEM16F control | | | TMEM16F Niclosamide | TMEM16F 1PBC |
|---|---|---|---|---|---|
| State | State B | Class 1 | Class 2 | | |
| EMDB | 41134 | 41137 | 41136 | 40776 | 40768 |
| PDB | 8TAG | 8TAL | 8TAI | 8SUR | 8SUN |
| **Data collection and processing** | | | | | |
| Microscope/ Detector | Titan Krios/Gatan K3 with Gatan Bioquantum energy filter | | | | |
| Imaging software and collection | SerialEM, 3x3 image shift | | | | |
| Magnification | 105,000 | | | | |
| Voltage (kV) | 300 | | | | |
| Electron exposure (e$^-$/Å$^2$) | 66 | | | | |
| Dose rate (e$^-$/pix/sec) | 8 | | | | |
| Frame exposure (e$^-$/Å$^2$) | 0.55 | | | | |
| Defocus range (µm) | (-0.8) - (-2.2) | | | | |
| Pixel size (Å) | 0.839 | | | | |
| Micrographs | 2526 (0º tilt)<br>6348 (30º tilt) | | | 4404 (0º tilt)<br>5359 (35º tilt)<br>5474 (45º tilt) | 2160 (0º tilt)<br>3062 (35º tilt) |
| **Reconstruction** | | | | | |
| Picked particles<br><br>(Blob picker in cryosparc) | | | | | |
| Particles in final refinement | 322,295 | 426,618 | 539,866 | 250,553 | 203,646 |
| Symmetry imposed | C1 | C1 | C1 | C1 | C1 |
| Guinier plot B factor (1/Å$^2$) | 152.1 | 158.8 | 158.9 | | 135.8 |
| Map resolution, global FSC (Å) | | | | | |
| FSC 0.143, unmasked/masked | 3.7/3.2 | 3.7/3.2 | 3.5/3.1 | 3.3/3.1 | 3.6/2.9 |
| **Refinement** | | | | | |
| Initial model used, PDB code | 6P48 | | | | |
| Model resolution (Å) | | | | | |
| FSC 0.5 unmasked/masked | 3.5/3.4 | 3.4/3.3 | 3.7/3.5 | 3.4/3.3 | 3.6/3.5 |
| Model composition | | | | | |
| Non-hydrogen atoms | 23599 | 23599 | 23599 | 23793 | 23624 |
| Protein residues | 1448 | 1448 | 1448 | 1448 | 1448 |
| Glycans (NAG) | 6 | 6 | 6 | 12 | 6 |
| *B* factors (Å$^2$) | | | | | |
| Protein | 193.10 | 79.69 | 76.03 | 143.92 | 88.38 |
| Ligand | 197.07 | 79.85 | 80.12 | 152.33 | 80.69 |
| R.m.s. deviations | | | | | |
| Bond lengths (Å) | 0.004 | 0.003 | 0.004 | 0.004 | 0.003 |
| Bond angles (º) | 0.712 | 0.547 | 0.589 | 0.776 | 0.572 |
| Validation | | | | | |
| MolProbity score | 1.47 | 1.72 | 1.85 | 1.41 | 1.80 |
| Clashscore | 4.07 | 6.97 | 5.65 | 3.95 | 6.13 |
| Poor rotamers (%) | 0 | 0 | 0 | 0 | 0 |
| EMRinger score | 2.08 | 3.00 | 2.41 | 3.08 | 1.92 |
| CaBLAM score | 2.35 | 1.99 | 1.99 | 1.57 | 1.85 |
| Ramachandran plot | | | | | |
| Favored (%) | 96.00 | 95.79 | 95.37 | 96.49 | 95.23 |
| Allowed (%) | 3.93 | 4.21 | 4.49 | 3.30 | 4.63 |
| Disallowed (%) | 0.07 | 0.00 | 0.14 | 0.21 | 0.14 |

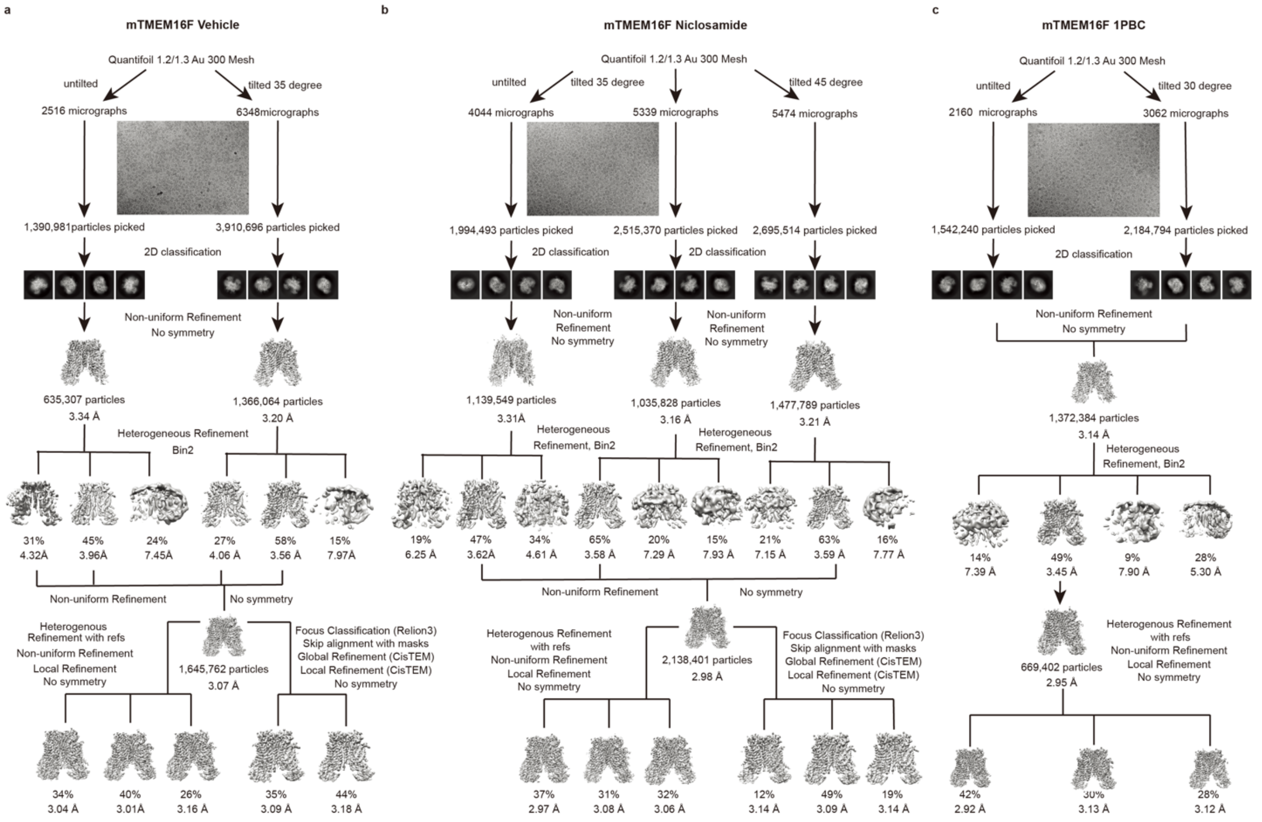


**Supplementary Figure 1. Cryo-EM workflow.** Representative micrographs and processing pipeline for TMEM16F purified in the absence of inhibitors (**a**) or in the presence of (**b**) niclosamide or (**c**) 1PBC. Briefly, micrographs were collected at different tilting angles and CTF correction was performed using CTF Patch in cryoSPARC. Particles were picked using Blobpicker, followed by 2D classification and Non-uniform refinement. Several rounds of consecutive Heterogenous refinement and non-uniform refinement rendered 3 high resolution classes (States A, B and C). Particles were subjected to focused classification without alignment in Relion 3.1 using a mask around the TM1-TM6 groove and the resulting classes underwent a final round of C1 refinement in cisTEM.

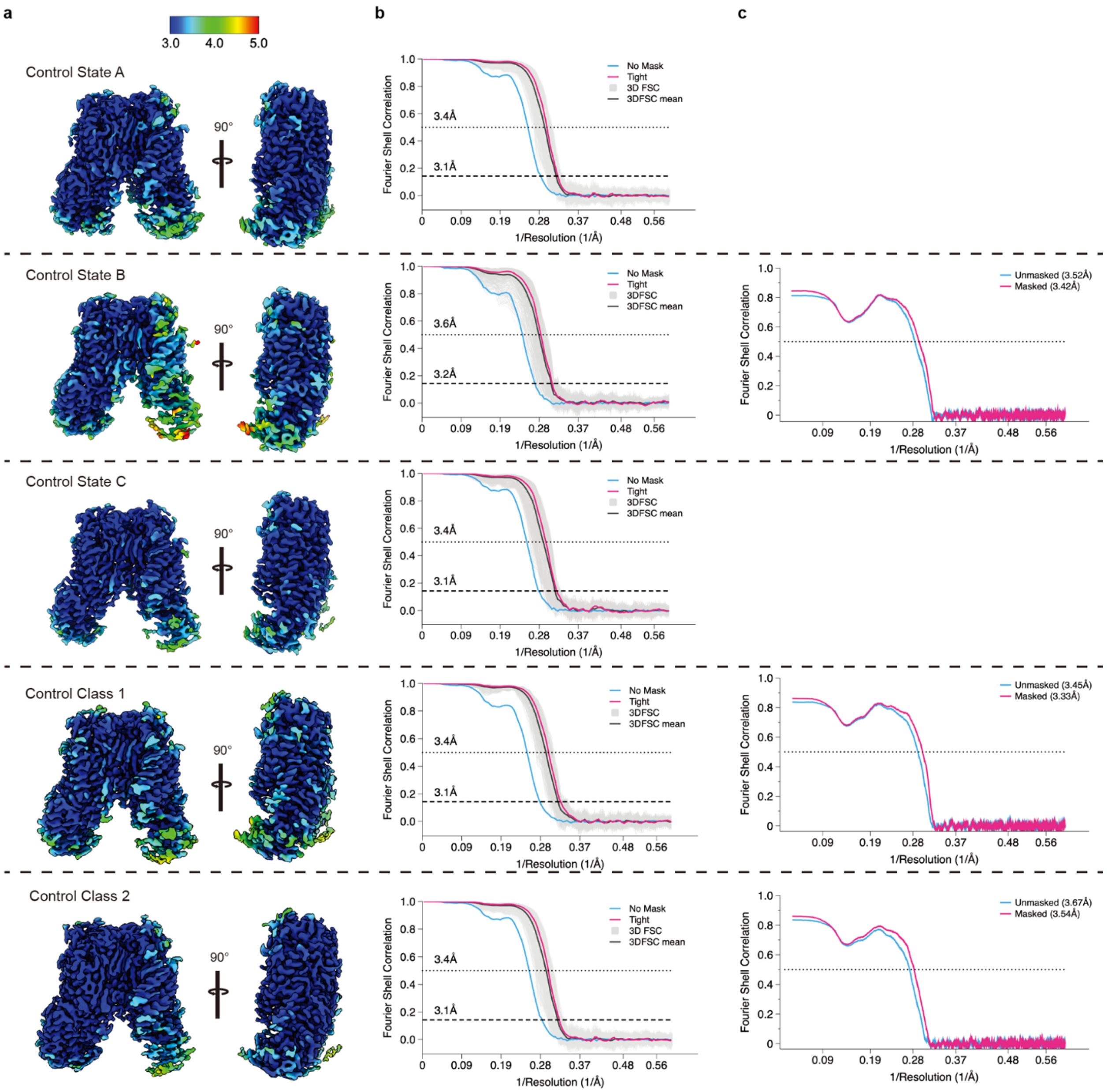


**Supplementary Figure 2. Validation of cryo-EM maps and models of unliganded TMEM16F.** **a** Local resolution estimates of TMEM16F reconstructions as reported by cryoSPARC with all maps colored on the same scale, as indicated. **b** Golden Standard Fourier Shell Correlation (GSFSC) and 3DFSC plots for cryo-EM maps. Resolution values at FSC=0.143 and 0.5 are noted. (**c**) Model-map correlation calculated in Phenix with resolution values at FSC= 0.5 in parenthesis.

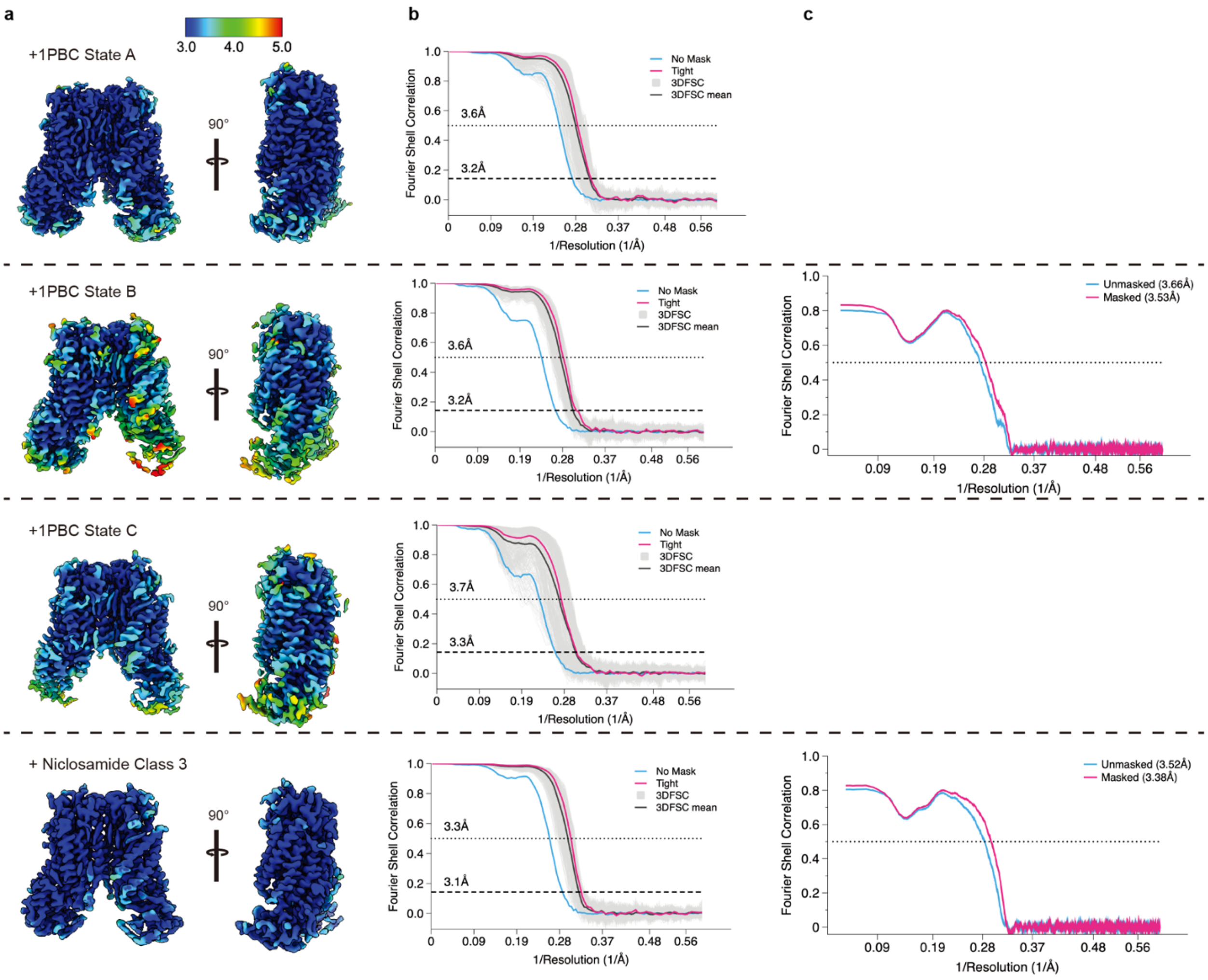


**Supplementary Figure 3. Validation of cryo-EM maps and models of TMEM16F in the presence of inhibitors. a** Local resolution estimates of TMEM16F reconstructions as reported by cryoSPARC with all maps colored on the same scale, as indicated. **b** Golden Standard Fourier Shell Correlation (GSFSC) and 3DFSC plots for cryo-EM maps. Resolution values at FSC=0.143 and 0.5 are noted. **c** Model-map correlation calculated in Phenix with resolution values at FSC= 0.5 in parenthesis.

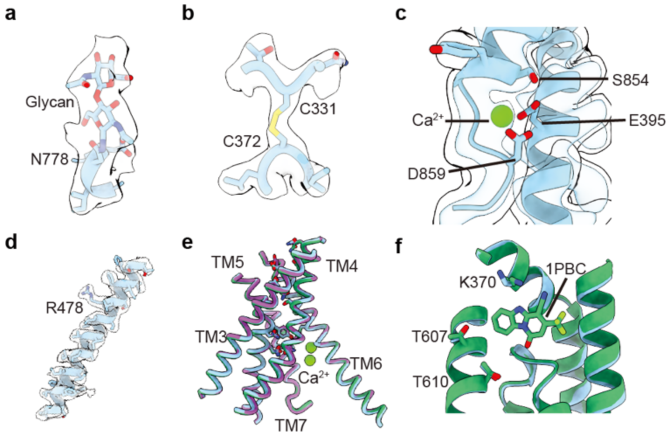


**Supplementary Figure 4. Structural details of TMEM16F.** Atomic model for (**a**) N-glycosylation at N778, (**b**) disulfide bond between residues C331 and C373, (**c**) third $Ca^{2+}$ binding site at the dimer interface and (**d**) TM4. In all cases, the cryo-EM density is shown in semitransparent and the residues are colored by heteroatom. Overlay of the ion conduction channel (**e**) and the drug binding site (**f**) in unliganded (blue), niclosamide- (purple) and 1PBC-bound (green) structures of TMEM16F.

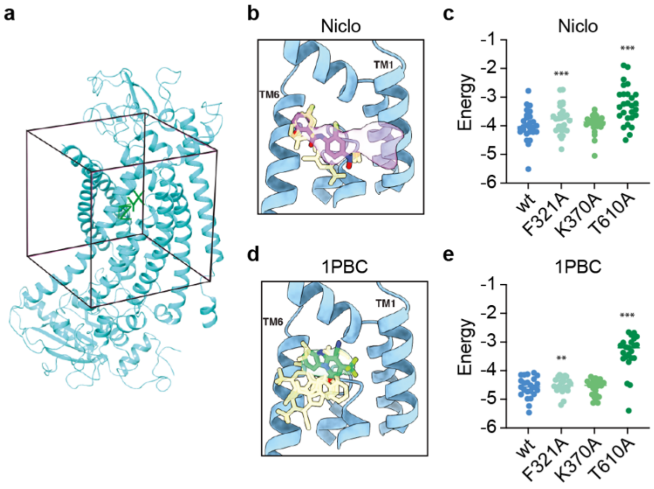


**Supplementary Figure 5. Computational docking of niclosamide and 1PBC in TMEM16. a** The Glide software was used to computationally dock niclosamide and 1PBC into the boxed area in the atomic model of TMEM16F. The top 5 ranking poses of niclosamide (**b**) and 1PBC (**d**) are shown in yellow with the best fitting position highlighted in purple and green, respectively. Comparison of the top ranked inhibitor poses by Glide in TMEM16F wildtype and alanine mutations, niclosamide (Niclo) (**c**) n = 27 in each group, 1PBC (**e**) n = 23 in each group. Statistical significance determined by paired t-test (two-tailed) for each mutant as compared to wildtype controls (F321A: $p < 0.0001$; K370A: $p = 0.2312$; T610A: $p < 0.0001$ in **(c)** and F321: $p = 0.0026$; K370A: $p = 0.9295$; T610A: $p < 0.0001$ in **(e)**, (*$p < 0.05$; **$p < 0.01$; ***$p < 0.001$).

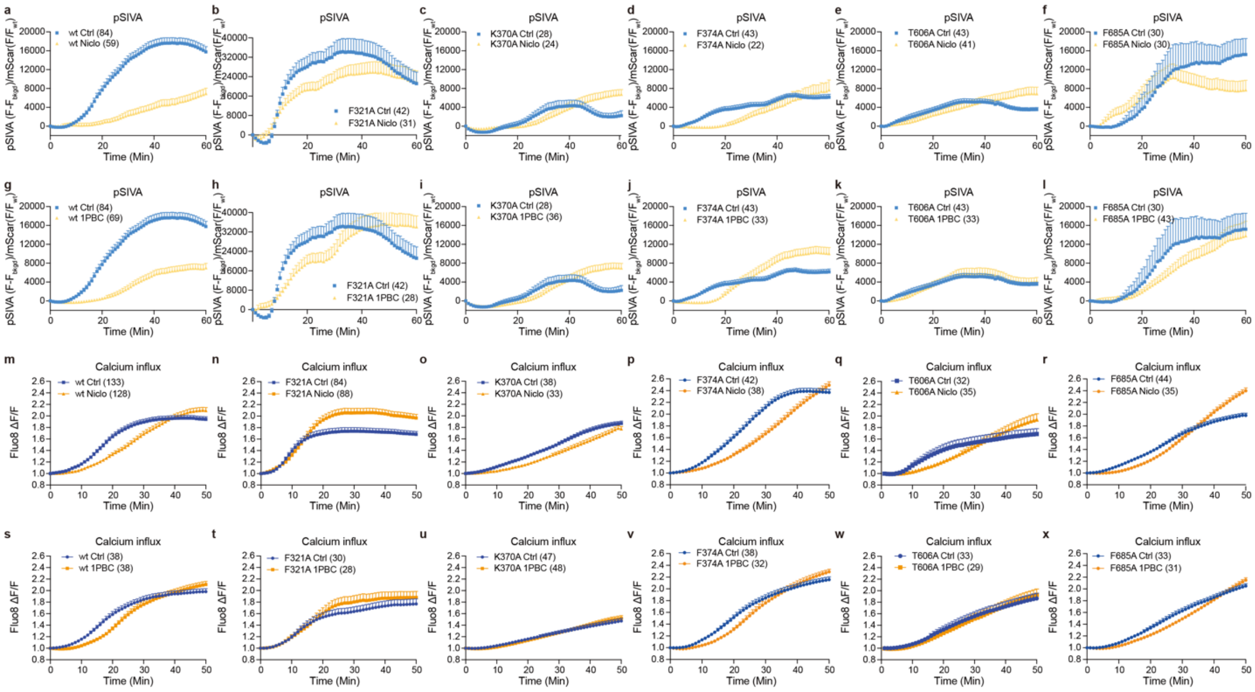


**Supplementary Figure 6. Functional validation for TMEM16F**. Representative curves of live imaging of TMEM16F-dependent PS exposure (**a** - **l**) and $Ca^{2+}$ influx (**m** - **x**). The pSIVA time courses are complex because after chemical induction of TMEM16F activation that causes extracellular vesicle release, some cells may eventually either undergo apoptosis or rupture, causing pSIVA to reach intracellular membranous compartments. Hence we focus our analyses on the latency for onset of PS exposure and $Ca^{2+}$ influx (Fig. 4). Data are represented as mean ± SEM. At least three independent experiments have been performed for each condition. Number of experiments (n) is given in parenthesis.

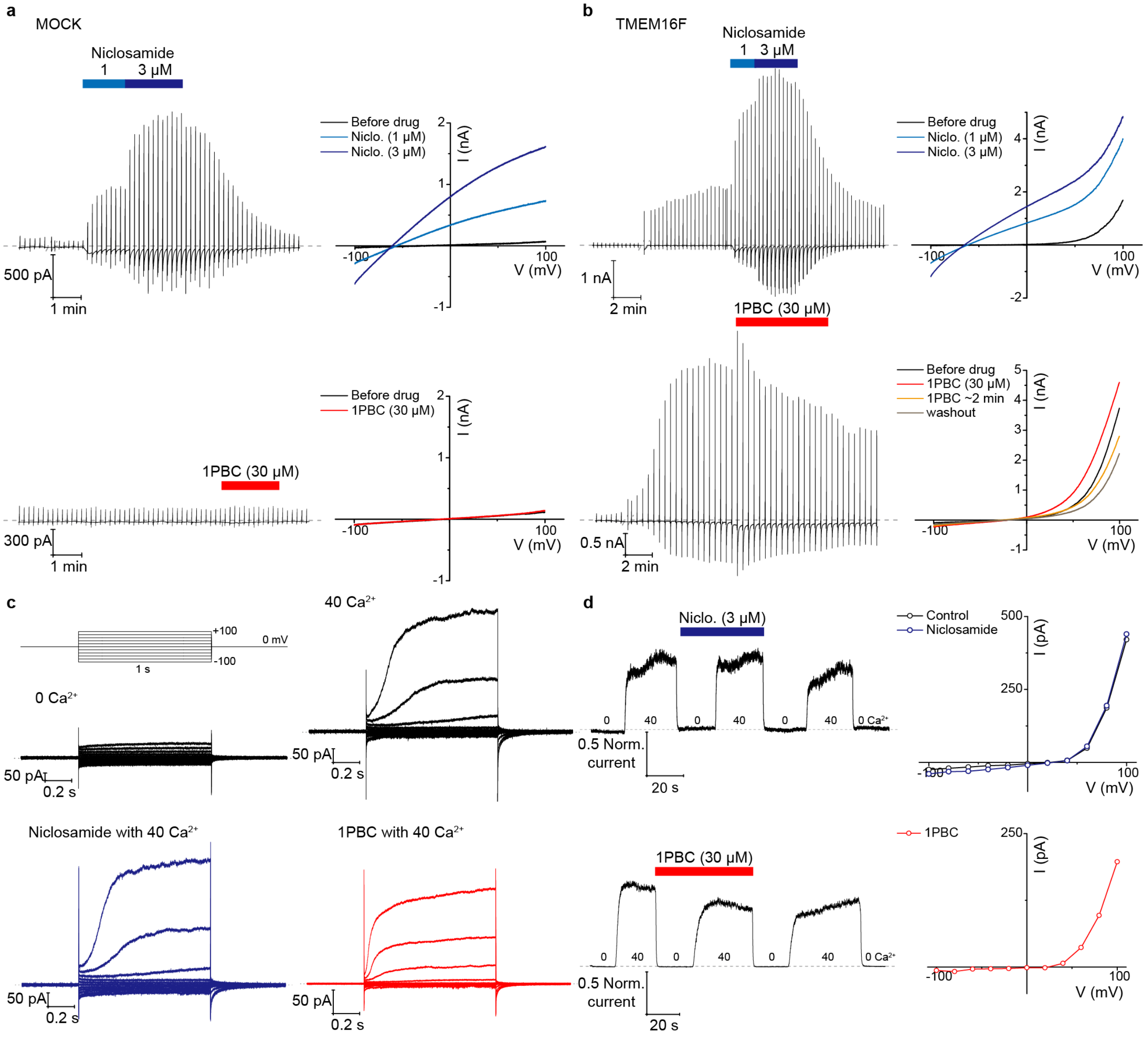


**Supplementary Figure 7. Whole-cell and inside-out patch recordings of TMEM16F currents. (a-b)** Representative whole-cell current trace and I-V curve of control cells with mock transfection (**a**) and cells expressing TMEM16F (**b**), treated with 1 and 3 µM niclosamide (top row) or 30 µM 1PBC (bottom row) when the cells reached the peak current, about 7 min after making whole cell configuration. The cells were recorded at 0 mV holding potential with the ramp pulse protocol from -100 to +100 mV for 1 s every 10 s. Colored lines of I-V curve indicate the time point at the end of each drug treatment. The number of experiments (n): control cells with MOCK-transfection treated with niclosamide (n = 7); cells expressing TMEM16F treated with niclosamide (n = 7); control cells with MOCK-transfection treated with 1PBC (n = 6); cells expressing TMEM16F treated with 1PBC (n = 10) **(c-d)** Representative inside-out patch recordings of TMEM16F current with niclosamide or 1PBC application to the patch. **c** Current trace obtained by step pulse protocol in different conditions; without calcium; with 40 µM internal calcium; 3 µM niclosamide with 40 µM calcium; 30 µM 1PBC with 40 µM calcium. **d** Representative current trace from excised patch recordings at +60 mV holding potential. The calcium concentration is indicated at the bottom of

the trace. Corresponding I-V curve is plotted with steady-state currents measured from -100 to +100 mV with 20 mV step pulse intervals. The number of experiments (n): cells expressing TMEM16F with inside-out patch exposed to niclosamide (n = 10); cells expressing TMEM16F with inside-out patch exposed to 1PBC (n = 7).

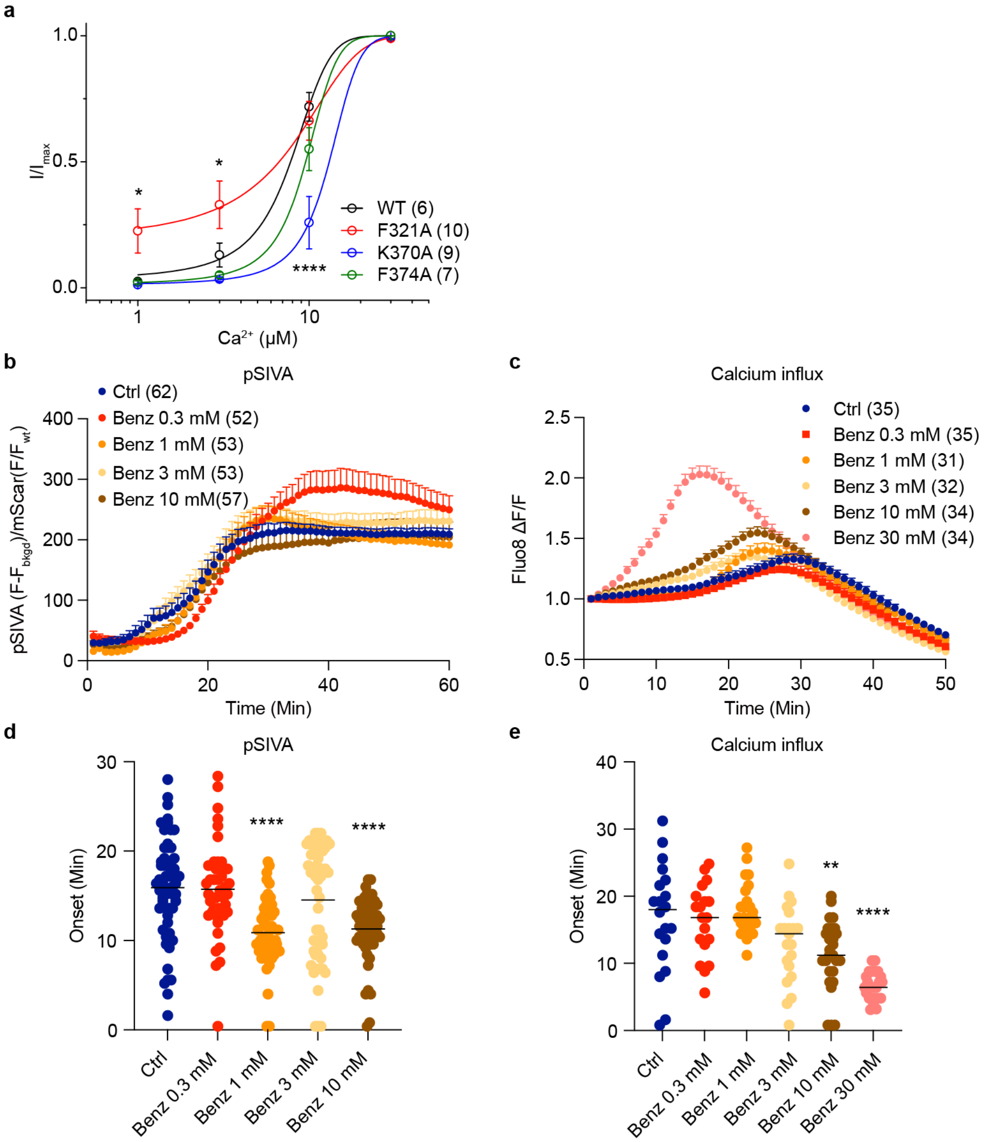


**Supplementary Figure 8. $Ca^{2+}$ sensitivity of F321A, K370A and F374 A mutant TMEM16F currents and the effect of benzbromorone on TMEM16F-mediated $Ca^{2+}$ influx and PS exposure. a.** $Ca^{2+}$-sensitivity curve. Normalized currents fit to the Hill equation. The mean ± SEM is shown along with the statistical significance determined by two-way ANNOVA (1 μM [$Ca^{2+}$]: WT *vs* F321A, p = 0.343; 3 μM [$Ca^{2+}$]: WT *vs* F321A, p = 0.366; 10 μM [$Ca^{2+}$]: WT *vs* K370A, $p < 0.0001$). Representative curves of live imaging of TMEM16F-dependent PS exposure (**b**); and $Ca^{2+}$ influx (**c**). Data are represented as mean ± SEM. Scattered dot plots of time of onset of TMEM16F-dependent PS exposure (**d**) n = 57 for Ctrl; 46 for Benz 0.3 μM; 52 for Benz 1 μM; 53 for Benz 3 μM; 56 for Benz 10 μM and $Ca^{2+}$ influx (**e**) n = 20 for Ctrl; 19 for Benz 0.3 μM; 26

for Benz 1 µM; 21 for Benz 3 µM; 27 for Benz 10 µM; 24 for Benz 30 µM. Time of onset could not be determined for time courses with an apparently linear rather than sigmoidal rise. At least three independent experiments have been performed for each condition. The number of experiments (n) is given in parenthesis. The mean ± SEM is shown along with the statistical significance determined by unpaired t-test (two-tailed) for each mutant as compared to vehicle controls (Benz 0.3 µM: $p = 0.8581$; Benz 1 µM: $p < 0.0001$; Benz 3 µM: $p = 0.2292$; Benz 10 µM: $p < 0.0001$ in **(d)** and Benz 0.3 µM: $p = 0.8345$; Benz 1 µM: $p = 0.5374$; Benz 3 µM: $p = 0.0857$; Benz 10 µM: $p = 0.0059$; Benz 30 µM: $p < 0.0001$ in **(e)**, *$p < 0.05$; **$p < 0.01$; ***$p < 0.001$; ****$p < 0.0001$).

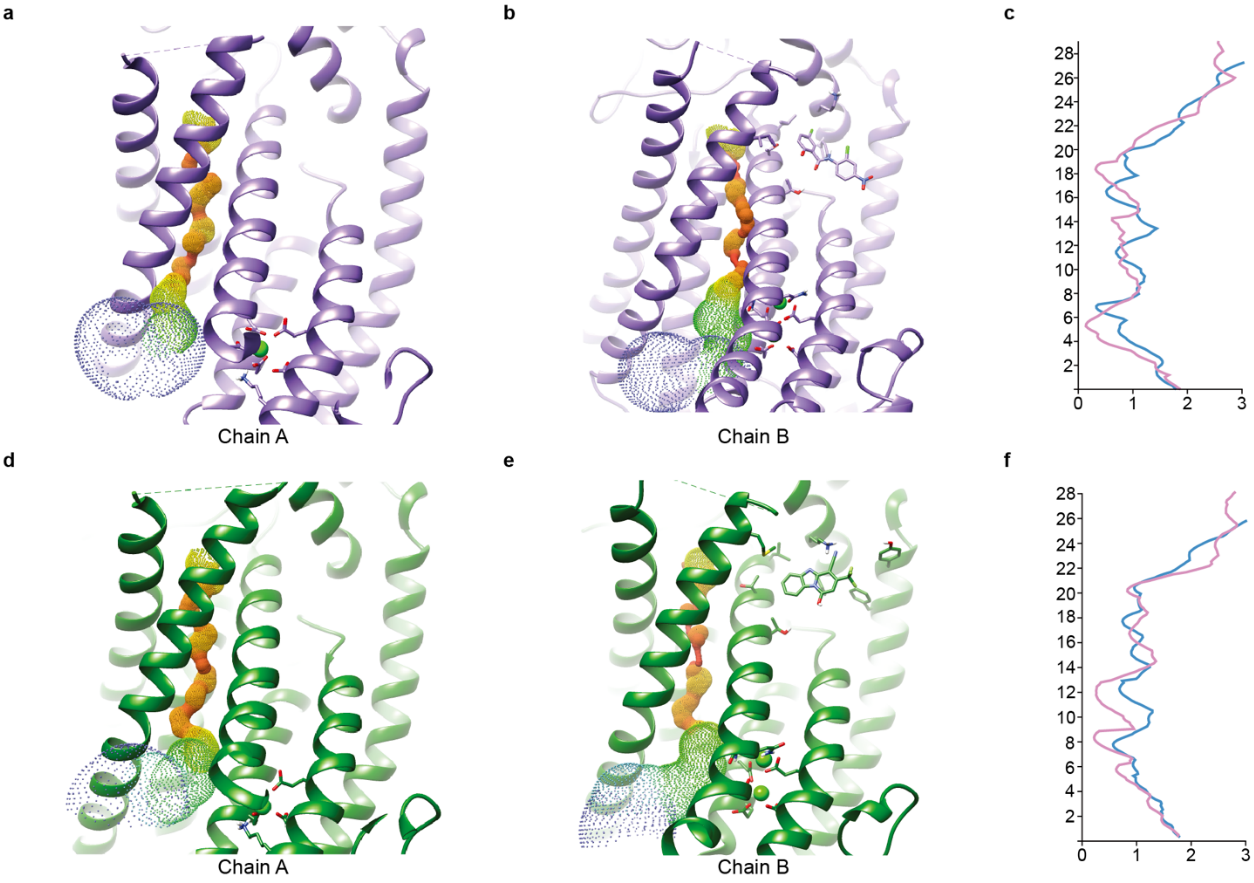


**Supplementary Figure 9. The pores of TMEM16F in ligand binding structures.** The solvent-accessible (mesh) pore of TMEM16F in $PIP_2$-supplemented nanodiscs with niclo (**a** and **b**) or 1PBC application (**d** and **e**). Calcium ions are shown in green. Pore radius along the z-axis for TMEM16F with niclosamide (**c**) and TMEM16F with 1PBC (**f**), blue for Chain A and pink for Chain B.
