# Supplementary material for: Identification of a drug binding pocket in TMEM16F calcium-activated ion channel and lipid scramblase
Source: Nat Commun. 2023 Aug 12;14:4874. doi: 10.1038/s41467-023-40410-x (PMC10423226; doi:10.1038/s41467-023-40410-x)
Supplement: Supplementary file 2 — Reporting Summary [file 41467_2023_40410_MOESM2_ESM.pdf]

nature portfolio

Corresponding author(s): Yifan Cheng and Lily Jan

Last updated by author(s): Jun 29, 2023

Reporting Summary

Nature Portfolio wishes to improve the reproducibility of the work that we publish. This form provides structure for consistency and transparency in reporting. For further information on Nature Portfolio policies, see our Editorial Policies and the Editorial Policy Checklist.

Statistics

For all statistical analyses, confirm that the following items are present in the figure legend, table legend, main text, or Methods section.

n/a	Confirmed	
☐	☒	The exact sample size (*n*) for each experimental group/condition, given as a discrete number and unit of measurement
☐	☒	A statement on whether measurements were taken from distinct samples or whether the same sample was measured repeatedly
☐	☒	The statistical test(s) used AND whether they are one- or two-sided<br>*Only common tests should be described solely by name; describe more complex techniques in the Methods section.*
☐	☒	A description of all covariates tested
☐	☒	A description of any assumptions or corrections, such as tests of normality and adjustment for multiple comparisons
☐	☒	A full description of the statistical parameters including central tendency (e.g. means) or other basic estimates (e.g. regression coefficient) AND variation (e.g. standard deviation) or associated estimates of uncertainty (e.g. confidence intervals)
☐	☒	For null hypothesis testing, the test statistic (e.g. *F*, *t*, *r*) with confidence intervals, effect sizes, degrees of freedom and *P* value noted<br>*Give P values as exact values whenever suitable.*
☒	☐	For Bayesian analysis, information on the choice of priors and Markov chain Monte Carlo settings
☒	☐	For hierarchical and complex designs, identification of the appropriate level for tests and full reporting of outcomes
☒	☐	Estimates of effect sizes (e.g. Cohen's *d*, Pearson's *r*), indicating how they were calculated

Our web collection on statistics for biologists contains articles on many of the points above.

Software and code

Policy information about availability of computer code

Data collection	Images were recorded using SerialEM 3.8 in super-resolution mode, followed by on-the-fly motion correction by MotionCor 2 1.3.1.
Data analysis	CryoSPARC 3.1.0 was used for particle picking and 2D classification. RELION3.1 and cisTEM 1.0.0-beta was used for 3D classification, and refinement procedures. COOT 0.9.4 and PHENIX 1.19 were used for model building. UCSF Chimera 1.14 and UCSF ChimeraX1.3 were used for image and map analysis. Graphpad Prism 8 was used for data processing of functional assays.

For manuscripts utilizing custom algorithms or software that are central to the research but not yet described in published literature, software must be made available to editors and reviewers. We strongly encourage code deposition in a community repository (e.g. GitHub). See the Nature Portfolio guidelines for submitting code & software for further information.

Data

Policy information about availability of data

All manuscripts must include a data availability statement. This statement should provide the following information, where applicable:

- Accession codes, unique identifiers, or web links for publicly available datasets
- A description of any restrictions on data availability
- For clinical datasets or third party data, please ensure that the statement adheres to our policy

Data and Code Availability
The accession numbers of cryo-EM density maps of TMEM16F reported in this paper are EMD: 41134 (https://www.ebi.ac.uk/pdbe/entry/emdb/EMD-41134),

41137 (https://www.ebi.ac.uk/pdbe/entry/emdb/EMD-41137), 41136 (https://www.ebi.ac.uk/pdbe/entry/emdb/EMD-41136), 40776 (https://www.ebi.ac.uk/pdbe/entry/emdb/EMD-40776) and 40768 (https://www.ebi.ac.uk/pdbe/entry/emdb/EMD-40768). The accession numbers of atomic coordinates for TMEM16F reported in this paper are PDB: 8TAG (https://doi.org/10.2210/pdb8TAG/pdb), 8TAL (https://doi.org/10.2210/pdb8TAL/pdb), 8TAI (https://doi.org/10.2210/pdb8TAI/pdb), 8SUR (https://doi.org/10.2210/pdb8SUR/pdb) and 8SUN (https://doi.org/10.2210/pdb8SUN/pdb). All electrophysiological data generated or analyzed during this study are included in the published article. All other data are available from the corresponding authors upon reasonable request.

Research involving human participants, their data, or biological material

Policy information about studies with human participants or human data. See also policy information about sex, gender (identity/presentation), and sexual orientation and race, ethnicity and racism.

Reporting on sex and gender	N/A
Reporting on race, ethnicity, or other socially relevant groupings	N/A
Population characteristics	N/A
Recruitment	N/A
Ethics oversight	N/A

Note that full information on the approval of the study protocol must also be provided in the manuscript.

Field-specific reporting

Please select the one below that is the best fit for your research. If you are not sure, read the appropriate sections before making your selection.

☒ Life sciences ☐ Behavioural & social sciences ☐ Ecological, evolutionary & environmental sciences

For a reference copy of the document with all sections, see nature.com/documents/nr-reporting-summary-flat.pdf

Life sciences study design

All studies must disclose on these points even when the disclosure is negative.

Sample size	Each dataset contains sufficient number of original micrographs. More than 1 million raw particles were selected using automated particle picking program. Three datasets were collected to determine the structures in this study. The number of micrographs and particles in each dataset is presented in Supplementary Table 1.
Data exclusions	No data were excluded.
Replication	No replication in data acquisition, which is not required for structural studies.
Randomization	Single particle cryo-EM data processing follows gold standard, that is to separate particle stacks by the order, even or odd, into two separate data sets, and processed separately. This separation is considered random. All biochemical experiments were initiated from multiple independent aliquots of the cells expressing the target proteins, and then subjected to indicated experimental conditions.
Blinding	All experiments are not blind. Blindness is not standard procedure in structural studies. The investigators need to know the target of the study for data processing.

Reporting for specific materials, systems and methods

We require information from authors about some types of materials, experimental systems and methods used in many studies. Here, indicate whether each material, system or method listed is relevant to your study. If you are not sure if a list item applies to your research, read the appropriate section before selecting a response.

Materials & experimental systems

n/a	Involved in the study
☒	☐ Antibodies
☐	☒ Eukaryotic cell lines
☒	☐ Palaeontology and archaeology
☒	☐ Animals and other organisms
☒	☐ Clinical data
☒	☐ Dual use research of concern
☒	☐ Plants

Methods

n/a	Involved in the study
☒	☐ ChIP-seq
☒	☐ Flow cytometry
☒	☐ MRI-based neuroimaging

Eukaryotic cell lines

Policy information about cell lines and Sex and Gender in Research

Cell line source(s)	Human embryonic kidney (HEK293 GnTi-) purchased from ATCC and Sf9 Insect Cells purchased from Expression Systems.
Authentication	None of the cell lines were authenticated
Mycoplasma contamination	Cell lines were not tested for mycoplasma contamination.
Commonly misidentified lines (See ICLAC register)	None
